# Previous Viral Infection Triggers Fungal Resistance Breakdown in Host Plants

**DOI:** 10.3390/pathogens15090973

**Published:** 2026-09-14

**Authors:** Nuraizat Abidin, Martin J. Barbetti, Ming Pei You, Roger A. C. Jones

**Affiliations:** 1 School of Agriculture and Environment, The University of Western Australia, Crawley, WA 6009, Australiamartin.barbetti@uwa.edu.au (M.J.B.); mingpei.you@uwa.edu.au (M.P.Y.); 2 UWA Institute of Agriculture, The University of Western Australia, Crawley, WA 6009, Australia

**Keywords:** *Brassica napus*, coinfection and pathogen interactions, *Leptosphaeria maculans*, polygenic resistance (PGR), compromised resistance and resistance-breaking strains, single dominant gene resistance (SDGR), temperature-sensitive systemic invasion resistance (TSSIR), turnip mosaic virus

## Abstract

Effects of viral and fungal coinfection upon host plant resistance effectiveness were studied using three canola (*Brassica napus*) cultivars with differing pathogen resistance characteristics, resistance-breaking (RB) and non-resistance-breaking (non-RB) *Leptosphaeria maculans* fungus strains, and turnip mosaic virus (TuMV) RB strain. These cultivars carried fungal single dominant gene resistance (SDGR), both fungal polygenic resistance and viral temperature-sensitive systemic invasion resistance (PGR + TSSIR), or no fungal resistance. Cotyledon viral RB strain inoculation was followed by two fungal spray inoculations onto systemically virus-infected plant foliage using different non-RB and RB strain combinations inoculated on the same day or 3 days apart. With PGR, fungal non-RB strain alone inoculation onto virus-infected foliage resulted in resistance breakdown from no infection to severe symptoms. For other fungal strain combinations, resistance breakdown was up to 7.4-fold with SDGR and 3.7-fold with PGR. TSSIR breakdown was absent following fungal inoculations onto virus-infected foliage. This study provides a model system that can not only improve understanding of virus–fungus pathogen interactions, but also identify where diminished single-pathogen host resistance effectiveness warrants reconsideration in coinfection scenarios that increase the likelihood of adverse economic impacts and where new cultivars with more effective fungal and viral pathogen host resistance are required.

## 1. Introduction

When epidemics caused by a single pathogen affect crops, pastures or natural ecosystems, other pathogens are often present on the same plants. Also, multiple pathogen complexes often cause epidemics. Coinfection and/or pathogen–pathogen interaction studies are therefore crucial to understanding the implications of such situations, as infection by another pathogen can compromise plant host resistance and increase both disease severity and pathogen incidence [1,2]. Moreover, resistance-breaking (RB) strains of plant pathogens [3,4,5] can occur amongst coinfections. Determining whether and how pathogen coinfections are likely to complicate epidemic outcomes, magnify disease-induced yield and quality losses and threaten plant-based industries or natural ecosystems is essential to the development of effective disease management strategies that address such situations [6,7,8].

Virus–fungus pathogen coinfection occurs commonly in plants ([9], references therein). A virus–fungus coinfection arising from both pathogens simply being present on the same plant is provided by two common oilseed canola (*Brassica napus*) pathogens, the fungus *Leptosphaeria maculans* and turnip mosaic virus (TuMV; genus *Potyvirus*). This fungus causes a disease called blackleg or Phoma stem canker [10], whereas this virus causes a leaf mosaic and plant stunting disease [11]. Canola cultivars often carry *L. maculans* and TuMV single-gene or polygenic (i.e., quantitative) resistances [11,12,13,14]. Both *L. maculans* and TuMV strains include canola RB and non-resistance-breaking (non-RB) strains [15,16,17]. Using canola cultivars with polygenic resistance (PGR) instead of single dominant gene resistance (SDGR) is recommended for suppressing *L. maculans* resistance breakdown by its RB strains [18,19,20]. Although widespread strain-specific viral SDGR in its cultivars formerly suppressed TuMV incidences in canola crops [12,21], an RB strain that overcomes all known TuMV single-gene resistances in cultivars tested so far now threatens the crop [9,17,22]. Moreover, although its first variants failed to overcome TuMV temperature-sensitive systemic invasion resistance (TSSIR), a variant that did this was found subsequently [16]. TSSIR restricts virus infection within leaves but is overcome when increased temperature allows systemic invasion to occur [9,22].

Abidin et al. [9] used: (i) canola cultivars with fungal SDGR, fungal PGR + viral TSSIR or without fungal resistance and (ii) RB fungal and viral strains and a non-RB fungal strain. Using virus inoculation with infective sap and fungus spore droplet inoculation, they found that cotyledon virus–fungus coinfection followed by systemic virus invasion produced different outcomes depending upon cultivar pathogen resistance characteristics, fungal strain combination and inoculation timing. Despite this progress, major knowledge gaps remain regarding: (1) whether viral suppression of host defenses can systematically compromise both single-gene and polygenic fungal resistance under realistic broad-surface inoculation conditions, (2) whether secondary fungal invasion can reciprocally overcome conditional viral resistance (such as TSSIR), and (3) how combinations of resistance-breaking and non-resistance-breaking pathogen strains interact in vivo. Addressing these gaps is critical to developing realistic disease models and safeguarding multi-gene host resistance in the field. Here, we hypothesized that spray inoculation with fungal spores would better simulate real-world field situations where fungal spores spread by wind and rain infect most of the foliage surface area [23,24]. Therefore, we undertook coinfection studies where a viral RB strain was inoculated to seedling cotyledons of cultivars with or without fungal SDGR or PGR or viral TSSIR. Foliage with or without systemic virus infection was sprayed twice on the same day or 3 days apart with different fungal RB and non-RB strain combinations. The aims were to determine: (i) whether previous infection would result in the virus acting as a ‘saboteur’ causing fungal host resistance (SDGR and PGR) breakdown after infection with different fungal RB and non-RB strain combinations; (ii) whether subsequent fungal infection would cause virus resistance (TSSIR) breakdown; and (iii) whether the approach adopted could provide a suitable model system for future foundational studies evaluating single-pathogen host plant resistance effectiveness when virus–fungus coinfection is present.

## 2. Materials and Methods

### 2.1. Plant Materials, Pathogen Strains and Growing Conditions

Canola cultivars and pathogen strain isolates employed previously by Abidin et al. [9] were used: Surpass 400 with fungal SDGR, Scoop with fungal PGR and viral TSSIR, and Westar lacking fungal resistance; RB strain isolates UWA192 (fungal) and 12.1 (viral); and non-RB strain isolate P11 (fungal). Isolate UWA192 overcomes canola fungal resistance gene *LepR3*, but isolate P11 does not [19,25,26,27]. Isolate 12.1 overcomes all known TuMV single-gene resistances in canola cultivars tested so far [16,17]. Also, despite TSSIR restricting this isolate to inoculated leaves at low temperatures, it was overcome at 22/18 °C day/night, allowing systemic invasion, although its virus concentration (VC) remained low [9].

Canola and Chinese cabbage (*B. rapa* subsp. *pekinensis* cv. Wombok) plants were kept in an air-conditioned glasshouse at 22/18 °C day/night temperatures under natural light conditions that included shading from bright direct sunlight. Canola seed was sown in square 9 × 9 × 18 cm pots, with cv. Westar seed being sown a week afterwards as it germinated one week earlier [9]. A standard potting mix was used, plants were hand watered daily, and Thrive™ liquid fertilizer (Yates Ltd., Padstow, New South Wales, Australia) was applied weekly following the manufacturer’s instructions.

### 2.2. Virus and Fungal Cultures and Inoculations

The virus culture was maintained via serial subculture in Chinese cabbage plants. Sap inoculation involved grinding TuMV-infected leaf material in extraction buffer [11.74 g L^−1^ dipotassium hydrogen phosphate (K_2_HPO_4_), 4.48 g L^−1^ potassium dihydrogen phosphate (KH_2_PO_4_) and 1.26 g L^−1^ sodium sulphite (Na_2_SO_3_)] mixed with abrasive (celite) and gently rubbing the infective sap onto Chinese cabbage leaves or canola cotyledons. For virus mock-inoculations, cotyledons were inoculated similarly but with healthy leaf extract.

Fungal inoculum was prepared as in previous studies (e.g., [9,19,25,26,27]). This involved reviving cultures from lyophilized ampule storage and culturing them on V8 agar plates until mature pycnidia appeared. Then, 0.5 × 1 cm agar strips were cut out, vortexed in a 10 mL suspension, and spread across agar plates maintained at 22 °C for 1 week under a mixed white and UV black light environment to promote pycnidia production. Then 10 mL of deionized water was added to culture plates, and their surfaces were rubbed with a glass rod. The resulting conidial suspension was filtered using Miracloth (Calbiochem, San Diego, CA, USA), and the final concentration was adjusted to 10^7^ conidia mL^−1^ using a hemocytometer counting chamber (Paul Marienfeld GmbH & Co., Lauda-Königshofen, Germany). Tween-20 (0.01%) was added to the conidial suspension to ensure its retention on plant surfaces. For fungus mock-inoculation, deionized water was used instead. Fungal inoculation to plants was undertaken under humid glasshouse conditions.

Canola cotyledons were inoculated with virus isolate 12.1 or virus mock-inoculated 15 (Westar) or 21 (Surpass 400, Scoop) days after sowing. Next, the plants were grown to the 1.07 Sylvester-Bradley Growth Stage (SBGS) [28] when the first fungal spray inoculation, or deionized water spray for fungus mock-inoculation, was applied onto the entire plant foliage at 50 days after virus cotyledon inoculation. This involved thorough spraying, including both adaxial and abaxial leaf surfaces and stems, until surface run-off occurred after which the plants were incubated for 96 h in clear plastic boxes to maintain high humidity favorable for fungal disease development. The second spray inoculation was repeated on the same day (i.e., 0–0 dai) or after 3 days (i.e., 0–3 dai). These two specific double inoculation sequences were used previously by Abidin et al. [9]. The fungal strain combinations used were: (i) two with the same RB or non-RB strain used twice, (ii) four with all possible first and second RB and non-RB strain combinations, and (iii) one consisting of a 50:50% combined RB and non-RB inoculum strain mixture used twice.

### 2.3. Virus Detection, Concentration and Symptom Intensity

At 45 days after virus inoculation or virus mock-inoculation, a sample consisting of non-necrotic leaf tissue was taken from a newly emerged leaf on each plant. This process was repeated after 58–59 or 61–62 days, i.e., 5 days before the first fungal spray inoculation and at 13–14 (i.e., 0–0 dai) or 16–17 (i.e., 0–3 dai) days after the second fungal inoculation. Each leaf was kept in a sealed plastic bag placed within a cooler box. Within 24 h, double-antibody sandwich ELISA [29] was undertaken using Agdia Pathoscreen Test Kits (Agdia Inc., Elkhart, IN, USA) following the manufacturer’s instructions. Leaf tip samples from virus-inoculated plants or virus mock-inoculated plants were tested individually or in groups of four, respectively. Optical density (OD) absorbance values were measured in a microtiter plate reader (Bio-Rad model 680; Bio-Systems International, Perth, Western Australia, Australia), with A_405_ nm values at least twice those of extracts from healthy plants being considered TuMV positive. The A_405_ nm readings were then exponentially transformed (*e^x^*) to provide VC values based on antigen accumulation [9,30,31]. At the same time (45 days), as the leaf samples were taken prior to the first fungal inoculation, virus symptom intensity (SI) assessments were made for each individual plant using the 0–5 rating system of Abidin et al. [9]. These SI values were used to compare with the corresponding VC values also from before fungal inoculation. SI values could not be assessed after fungal inoculation because necrotic fungal symptoms obscured virus symptoms.

### 2.4. Fungal Disease Assessments

Fungal disease assessments were made on individual plants 11 days after the second fungal inoculation, with those for days 0 and 3 being assessed 3 days apart. These assessments measured leaf disease incidence (LDI) quantified as percentage of leaves showing disease symptoms (%LDI), leaf area diseased (LAD) quantified as proportion of infected leaf area (%LAD), and leaf area collapsed (LAC) quantified as proportion of collapsed leaf area (%LAC) [32]. The same researcher undertook all fungus disease scoring according to standard practice so there was no observer bias.

### 2.5. Experimental Design and Statistical Analysis

The experiment was duplicated. Each consisted of three canola cultivars x six fungus (five fungal strain/isolate combinations and fungus mock-inoculated) x two virus (systemically virus-infected and virus mock-inoculated) x two different inoculation times (0–0 and 0–3 dai) x eight replications/treatment (1 pot/replicate) (Table 1). The total number of pots was 576 per experiment, and each experiment was arranged in a randomized block design. Prior to the statistical analyses, the %LDI, %LAD, %LAC and *e^x^* transformed VC data were tested for normality and homogeneity of variance by both *t*-tests and F-tests, which they fully satisfied. Since data for the two experimental data sets were not significantly different (*p* > 0.05), both were combined and re-analyzed as a single data set. Analysis of variance (ANOVA) was performed in GenStat 23rd edition (VSN International Ltd., Hemel Hempstead, Hertfordshire, UK). Since Tukey’s Honestly Significant Difference (HSD) increases Type II errors (i.e., failure to detect actual differences), Fisher’s LSD was used instead in our factorial analyses because it maximizes statistical power to detect subtle effects [33]. Since adopting this approach with a large factorial design can inflate the family-wise error rate (FWER) and increase the probability of false positives, to mitigate this potential drawback we utilized an F-test which provides standard protection against FWER inflation prior to LSD testing. To ensure the mathematical assumptions of GenStat’s ANOVA were preserved, these tests were performed after *e^x^* transformation of ELISA OD (A_405_) VC data values. Significant differences between treatments were identified using Fisher’s least significant differences (LSDs) at *p* < 0.05. As virus or fungus mock-inoculated plants always developed nil (i.e., 1.1) *e^x^*-transformed VC and 0% LDI, LAD and LAC values, these were excluded from the statistical analyses to avoid causing data bias.

## 3. Results

### 3.1. Overall Outcomes

In terms of %LDI, %LAD and %LAC values at 11 days after the second fungal inoculation, there were significant main treatment effects (all *p* < 0.001) for the three cultivars, the six isolate combinations, and the two second fungal inoculation times (0–0 or 0–3 dai), and with VC for systemic virus infection versus viral mock-inoculation at 12–13 days after the second fungal inoculation. There were also significant two-, three-, and four-way interactions (all *p* < 0.001) between all combinations of the above treatments. Furthermore, after fungal inoculation across all three cultivars at both 0–0 and 0–3 dai, systemically virus-infected foliage always developed significantly greater %LDI, %LAD and %LAC values than virus mock-inoculated plants (Table 2), and %LDI, %LAD and %LAC values were highly positively correlated [%LDI and %LAD (y = 0.8233x − 2.4581, R^2^ = 0.73), %LAD and %LAC (y = 0.7987x − 2.6099, R^2^ = 0.71) and %LDI and %LAC (y = 0.9151x + 3.5296, R^2^ = 0.81)] (Supplementary Table S1a–e).

VC and SI measurements made on systemically virus-infected foliage 5 days before the first fungal inoculation provided values of 31–33 (VC) and SI (4.0) where fungal resistance was absent (Westar), 26–27 (VC) and 3.2 (SI) for SDGR (Surpass 400) and 14–15 (VC) and 0.9 (SI) for PGR + TSSIR (Scoop) (Table 2). When TSSIR was present, the resulting low VC values only elicited asymptomatic infection or faint mosaic.

### 3.2. Effect of Virus Infection on Fungal SDGR Effectiveness

**Figure 1:**
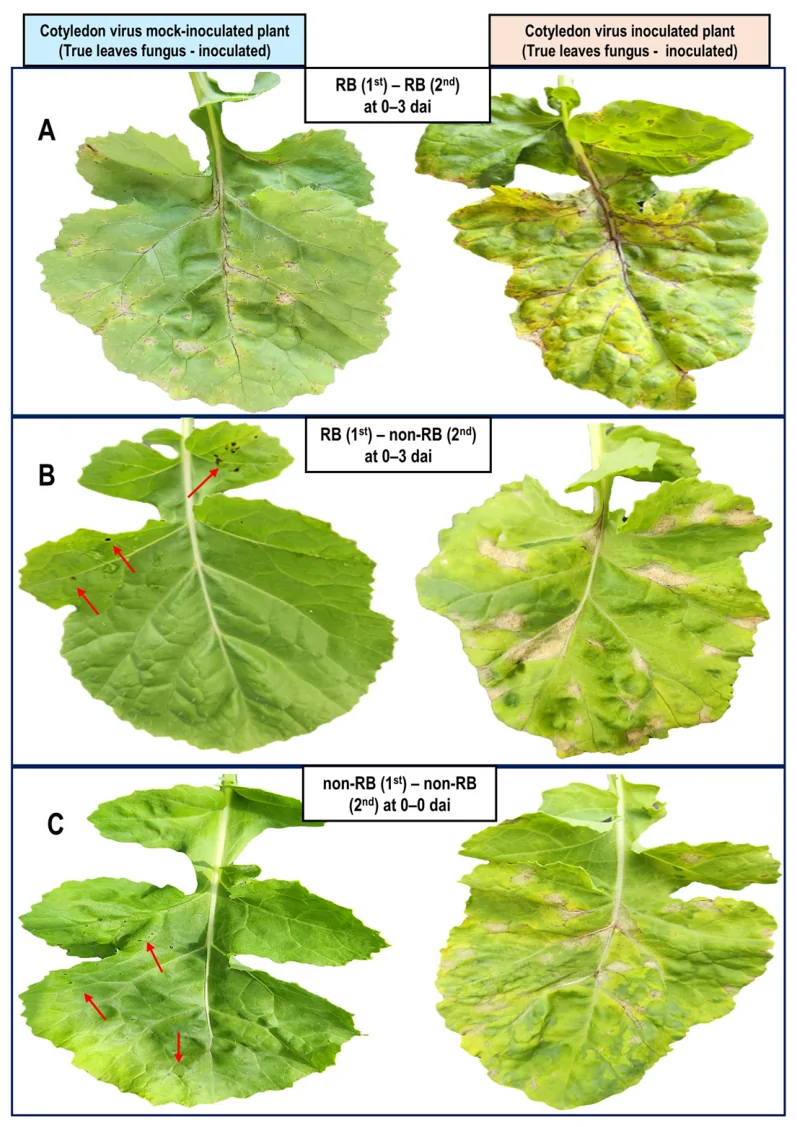
Paired leaf comparisons showing fungal (*Leptosphaeria maculans*) disease symptoms on true leaves from cotyledon mock-inoculated or virus-inoculated (turnip mosaic virus, TuMV) canola plants (cv. Surpass 400) carrying fungal single dominant gene resistance (SDGR). Mock-inoculated plant leaf (**left**) and systemically virus-infected leaf (**right**), both from foliage spray inoculated with fungal spores. Fungal disease foliage assessments were made 11 days after the second fungal inoculation, with those for same-day (0–0 dai) or 3-day (0–3 dai) inoculations assessed 3 days apart. Pathogen inoculations were with resistance-breaking (RB) strain isolates 12.1 (virus) and UWA192 (fungus) and non-resistance-breaking (non-RB) strain isolate P11 (fungus). Four different combinations of RB and non-RB fungal strains and a mixed RB and non-RB fungal strain inoculum were used. Red arrows show tiny necrotic lesions representing a Hypersensitive Response (HR) on inoculated leaves. (**A**) Large necrotic lesions with limited veinal necrosis (**left**) versus yellow mosaic and more extensive veinal necrosis (**right**) following first and second inoculations with fungal strain combination RB(1st)–RB(2nd) at 0–3 dai. (**B**) Small necrotic HR lesions (**left**) versus large, irregular-shaped spreading necrotic lesions and yellow mosaic (**right**) following first and second inoculations with fungal strain combination RB(1st)–non-RB(2nd) at 0–3 dai. (**C**) Tiny necrotic HR lesions (**left**) versus large, irregular-shaped spreading necrotic lesions and yellow mosaic (**right**) following first and second inoculations with fungal strain combination non-RB(1st)–non-RB(2nd) at 0–0 dai.

On virus-infected plants with SDGR, the combined inoculum mixture of both fungus RB and non-RB strains elicited values of %LDI 39, %LAD 41 and %LAC 39 at 0–0 dai and %LDI 65, %LAD 63% and %LAC 58 at 0–3 dai (Table 2). These values were significantly higher (*p* < 0.05) than those for all the other four strain combinations: LDI 33–34%, %LAD 34 and %LAC 30–32 at 0–0 dai and %LDI 52–53, %LAD 52–55 and %LAC 44–45 at 0–3 dai. By contrast, each fungal strain combination resulted in significantly smaller %LDI, %LAD and %LAC values on virus mock-inoculated than on virus-infected plants (Figure 1A–C). In virus mock-inoculated plants, these values were all significantly higher (*p* < 0.05) when the RB strain was present alone (%LDI of 26, %LAD 18% and %LAC 19 at 0–0 dai and %LDI 37, %LAD 22 and %LAC 27 at 0–3 dai) (Figure 1A) than with any of the other four strain combinations (%LDI + %LAD 10 and %LAC 6 at 0–0 dai and %LDI 10–16, %LAD 6–13 and %LAC 5–11 at 0–3 dai) (Figure 1B,C). Amongst these other four strain combinations, at 0–3 dai, the values for the RB strain combination RB (1st)–non-RB (2nd) for %LDI (16) and %LAD (13) were significantly higher (*p* < 0.05) than those for the other three. Thus, virus presence always caused considerable SDGR breakdown which was greatest when mixed fungal inoculum containing both non-RB and RB strains was used [fold increases of up to 3.7–6.3 (%LDI), 4.0–7.4 (%LAD) and both 6.2 (%LAC)]. Otherwise, they remained at lower levels following inoculation with different fungal strain combinations (Figure 1C). Without virus infection, SDGR breakdown only occurred when the fungal RB strain was present alone (Figure 1A).

### 3.3. Effect of Virus Infection on Fungal PGR Effectiveness

**Figure 2:**
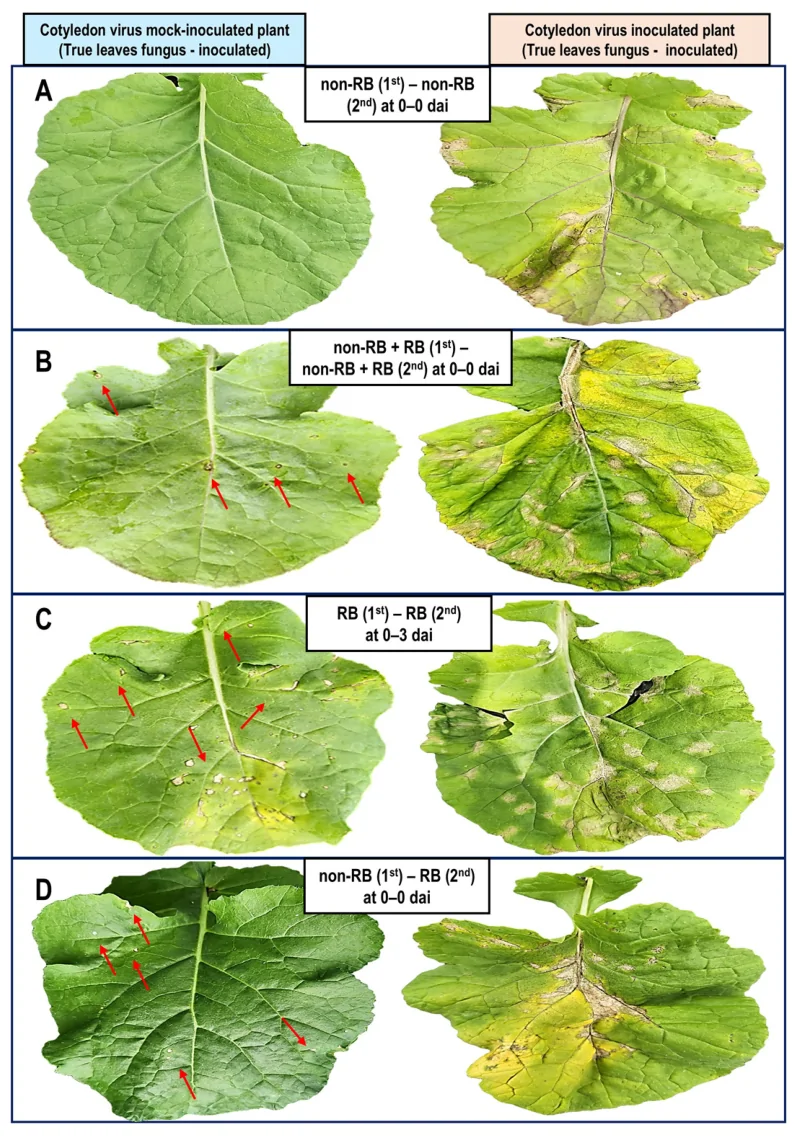
Paired leaf comparisons showing fungal (*Leptosphaeria maculans*) disease symptoms on true leaves from cotyledon mock-inoculated or virus-inoculated (turnip mosaic virus, TuMV) canola plants (cv. Scoop) carrying fungal polygenic resistance (PGR) and temperature-sensitive systemic invasion resistance (TSSIR). Mock-inoculated plant leaf (**left**) and systemically virus-infected leaf (**right**), both from foliage spray inoculated with fungal spores. Fungal disease foliage assessments were made 11 days after the second fungal inoculation, with those for same-day (0–0 dai) or 3-day (0–3 dai) inoculations assessed 3 days apart. Pathogen inoculations were with resistance-breaking (RB) strain isolates 12.1 (virus) and UWA192 (fungus) and non-resistance-breaking (non-RB) strain isolate P11 (fungus). Four different combinations of RB and non-RB fungal strains and a mixed non-RB and RB strain inoculum were used. Red arrows show tiny necrotic lesions. (**A**) Complete absence of any symptoms (**left**) versus extensive necrosis and mosaic (**right**) following first and second inoculations with fungal strain combination non-RB (1st)–non-RB (2nd) at 0–0 dai. (**B**) Tiny necrotic lesions (**left**) versus large, irregular-shaped spreading necrotic lesions with veinal necrosis and yellow mosaic (**right**) following first and second inoculations with mixed RB and non-RB strain inoculum at 0–0 dai. (**C**) Small necrotic lesions (**left**) versus large, irregularly shaped spreading necrotic lesions (**right**) following first and second inoculations with fungal strain combination RB (1st)–RB (2nd) at 0–3 dai. (**D**) Tiny necrotic lesions (**left**) versus large irregularly shaped necrotic lesions with extensively spreading veinal necrosis and yellow mosaic (**right**) following first and second inoculations with fungal strain combination non-RB (1st)–RB (2nd) at 0–0 dai.

On foliage of virus mock-inoculated plants with PGR + TSSIR, when the non-RB strain was present alone, the resulting %LDI, %LAD and %LAC values were all 0 following fungal inoculation at 0–0 dai and 0–3 dai (Table 2; Figure 2A). Infection with the other four fungal strain combinations resulted in values of %LDI 11–12, %LAD 11–12 and %LAC 10–11 at 0–0 dai and %LDI 18–23%, %LAD% 17–23, and %LAC 15–19 at 0–3 dai (Figure 2B–D). There were no statistically significant differences between the values for these four strain combinations at 0–0 dai. However, at 0–3 dai the value of 23% for %LAD was significantly higher (*p* < 0.05) where the RB strain was inoculated alone than the 0–17% values from when the non-RB strain was inoculated first. By contrast, at 0–0 and 0–3 dai, infection with each fungal strain combination always resulted in significantly higher (*p* < 0.05) %LDI, %LAD and %LAC values on virus-infected than on virus mock-inoculated plants (Figure 2A–D). The combined inoculum mixture of both fungus RB and non-RB strains resulted in significantly higher (*p* < 0.05) values (%LDI 42, %LAD 40, and %LAC 37 at 0–0 dai and %LDI of 57, %LAD of 56 and %LAC of 56m at 0–3 dai) than any of the other four strain combinations (Figure 2B). Also, when virus-inoculated plants were inoculated with the non-RB strain alone, this resulted in values of %LDI of 37, %LAD 35 and %LAC 30 at 0–0 dai and %LDI of 42, %LAD 43 and %LAC 42 at 0–3 dai, instead of the 0% values for all these parameters in virus mock-inoculated plants (Figure 2A). The three other strain combinations elicited values for %LDI of 34–38, %LAD of 34–35 and %LAC of 31–33 at 0–0 dai and %LDI of 43–51, %LAD of 43–49 and %LAC 41–49 at 0–3 dai. At 0–0 dai, the values for these three strain combinations were similar to those for the non-RB strain alone. By contrast at 0–3 dai, there were significant differences (*p* < 0.05) between these four strain combinations, where the two having RB strain first elicited significantly higher %LDI, %LAD and %LAC values than the two having the non-RB strain first. Thus, virus presence always overcame PGR, with this being most evident when the fungal inoculum mixture containing both non-RB and RB strains was used [fold increases of up to 3.0–3.7 (%LDI), 3.0–3.4 (%LAD) and 3.6–3.7 (%LAC)]. By contrast, there was no PGR suppression whatsoever when virus infection was absent and the non-RB strain was used alone, but partial PGR suppression with the other four strain combinations. When virus was present, however, the non-RB strain alone elicited statistically analogous levels of fungus disease to those caused by the other three strain combinations not involving mixed strain inoculum at 0–0 dai and to that of the non-RB first RB strain second combination at 0–3 dai. Since VC suppression was greatest when PGR and TSSIR were both present regardless of fungal strain combination, there was no evidence of TSSIR breakdown arising from fungus coinfection (see VC Section 3.5 below), so the PGR breakdown in virus-infected plants can be attributed mainly to virus coinfection.

### 3.4. Effect of Virus Infection on Fungal Disease Where Fungus Resistance Was Lacking

**Figure 3:**
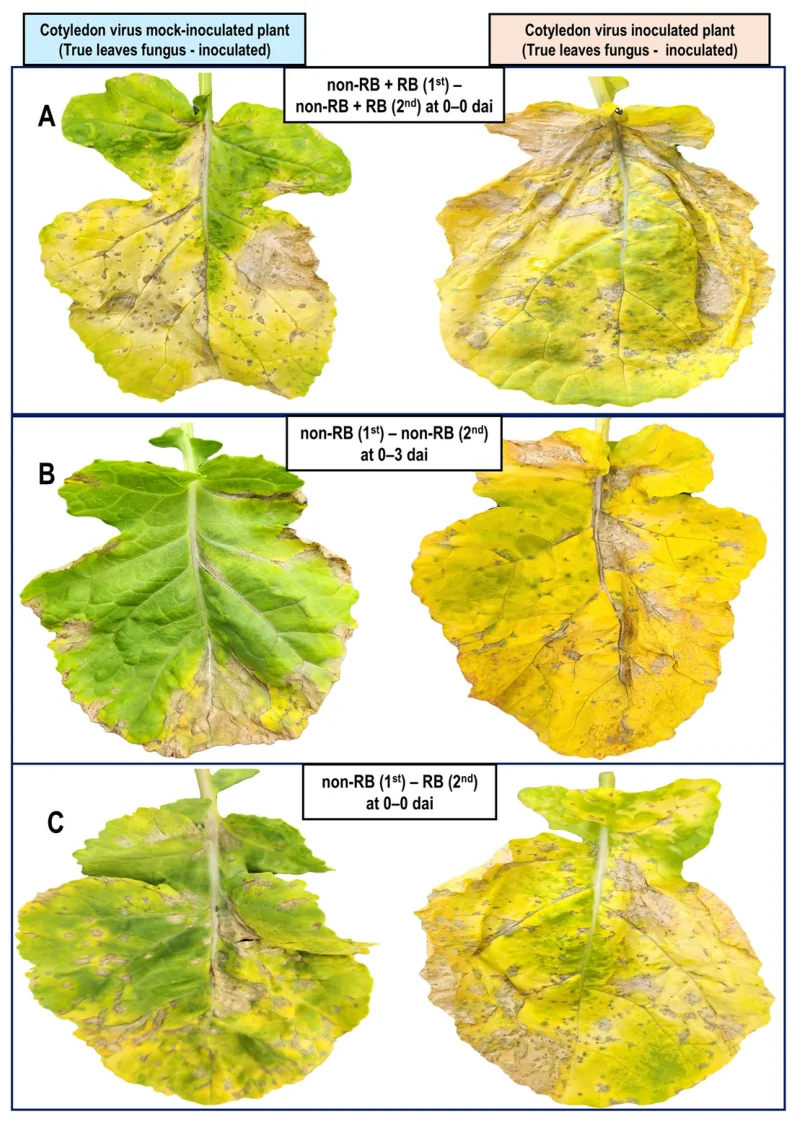
Paired leaf comparisons showing fungal (*Leptosphaeria maculans*) disease symptoms on true leaves from cotyledon mock-inoculated or virus-inoculated (turnip mosaic virus, TuMV) canola plants which lack any fungal resistances (cv. Westar). Mock-inoculated plant leaf (**left**) and systemically virus-infected leaf (**right**), both from foliage spray-inoculated with fungal spores. Fungal disease foliage assessments made 11 days after the second fungal inoculations, with those for same-day (0–0 dai) or 3-day (0–3 dai) inoculations assessed 3 days apart. Pathogen inoculations were with resistance-breaking (RB) strain isolates 12.1 (virus) and UWA192 (fungus) and non-resistance-breaking (non-RB) strain isolate P11 (fungus). Four different combinations of RB and non-RB fungal strains and a mixed non-RB and RB strain inoculum were used. (**A**) Irregular-shaped large spreading necrotic lesions and more generalized chlorosis, mosaic and necrotic areas (**left**) versus similar severe symptoms (**right**) following first and second inoculations with the same mixed non-RB and RB strain inoculum at 0–0 dai. (**B**) Irregular-shaped large necrotic areas, chlorosis and necrosis spreading along veins (**left**) versus similar but more extensive and severe symptoms (**right**) following first and second inoculations with fungal strain combination non-RB(1st)–non-RB(2nd) at 0–3 dai. (**C**) Large irregular necrotic lesions and spreading necrotic areas and yellow mosaic (**left**) versus similar but more severe symptoms plus leaf deformation (**right**) following first and second inoculations with fungal strain combination non-RB(1st)–RB (2nd) at 0–0 dai.

On foliage of virus-inoculated plants without fungal resistance, the combined inoculum mixture of both fungus RB and non-RB strains elicited increases of 10–14% (LDI), 14–17% (LAD) and 11–25% (LAC) (Table 2; Figure 3A). These increases were significantly higher (*p* < 0.05) than those for the other four fungal strain combinations (Figure 3B,C). Nevertheless, all five fungal strain combinations resulted in significantly smaller (*p* < 0.05) %LDI, %LAD and %LAC values in virus mock-inoculated than in virus-infected plants (Table 2). Thus, inoculation with fungal RB and non-RB strains either on their own or in different combinations always resulted in increases in fungal disease on systemically virus-infected plants. However, these increases were far smaller than those elicited when SDGR or PGR + TSSIR were present.

### 3.5. Effect of Fungus Infection on Virus Concentration Values and TSSIR Effectiveness

Compared with fungus mock-inoculated plant foliage, fungal inoculation onto systemically virus-infected foliage always resulted in statistically significant reductions (*p* < 0.05) in VC values regardless of whether SDGR, PGR + TSSIR or no fungal resistance were present (Table 2). However, its extent varied depending upon the fungal strain inoculum used, inoculation timing and whether SDGR or PGR + TSSIR were present or fungal resistance was lacking. With SDGR (Surpass 400), this suppression was from a VC value of 27.1 for fungal mock-inoculation. It was least when the fungal non-RB strain was present alone (VC reductions to 22.7 at 0–0 dai and 25.8 at 0–3 dai), compared with much greater suppression whenever the fungal RB strain was present (VC reductions to 15.2–15.4 at 0–0 dai and 13.3–15.3 at 0–3 dai). The greatest reduction was with the non-RB and RB strain inoculum mixture combination at 0–3 dai (VC reduction to 13.3). When PGR and TSSIR were both present, VC suppression from 14.6 was greatest with the non-RB and RB strain mixture combination (VC reductions to 7.8 at 0–0 dai and 4.1 at 0–3 dai) compared with the smaller decreases for the other fungal strain combinations, reductions to 8.3 at 0.0 dai or 6.3–6.4 at 0–3 dai (non-RB strain first) or 8.0–8.3 at 0–0 dai and 5.4–5.5 at 0–3 dai (RB strain first). The same VC suppression patterns at 0.0 and 0–3 dai applied to VC values in the newest tip leaves which were too small to inoculate when fungal inoculations were done. When fungal resistance was lacking, VC suppression from an initial value of 32.5 for fungal mock-inoculation varied considerably depending upon fungal strain combination. The greatest decreases occurred with the non-RB and RB strain inoculum mixture combination, with VC decreases to 14.3 at 0–0 dai and 9.8 at 0–3 dai, compared with the smaller decreases for the other fungal strain combinations, with declines to 17.8 at 0–0 dai and 14.2–14.3 at 0–3 dai (non-RB strain first) or 29.2–29.3 at 0–0 dai and 22.1–23.1 at 0–3 dai (RB strain first). VC values for newest tip leaves were highly positively correlated with those from the fungus-inoculated leaves (y = 1.99x + 6.01, *R*^2^ = 0.94). Thus, fungal inoculation onto systemically virus-infected foliage always resulted in statistically significant VC value reductions regardless of whether SDGR, PGR + TSSIR or no fungal resistance were present. However, its extent varied depending upon fungal second inoculation time and the fungal strain combination inoculated. With SDGR, VC suppression was least with the fungal non-RB strain alone combination and greatest with the non-RB and RB strain inoculum mixture. With PGR + TSSIR and when resistance was absent, VC suppression was also the greatest with this same combination. Since VC values were decreased to the greatest extent with PGR + TSSIR despite its overall fungal disease values being smaller than those with SDGR, there was no evidence of TSSIR breakdown arising from fungus coinfection [maximum VC decrease 49% for SDGR versus 72% for PGR + TSSIR].

## 4. Discussion

Here, we demonstrate that previous virus infection can cause fungal host resistance breakdown in coinfected plants. Spray inoculation with fungal pathogen spores onto systemically virus-infected foliage of crop cultivars differing in host resistance characteristics resulted in major breakdown of two crucial types of fungal host resistance, SDGR and PGR in the coinfected leaves. Also, varying the combinations of RB and non-RB strains of the fungal pathogen inoculated to systemically virus-infected foliage altered the extent of fungal resistance breakdown. Conversely, inoculating these same combinations of fungal strains onto systemically virus-infected foliage failed to break virus TSSIR. This novel finding of fungal pathogen resistance breakdown arising from previous viral infection seems not to have been reported before, yet it carries significant implications for scenarios reliant on such resistances. Moreover, our experimental system is unique because it shifts from a more artificial approach (cotyledon droplet fungal inoculation) to a more realistic field-simulating model (foliage spray fungal inoculation) which mimics how fungal spores spread via wind and rain across the entire foliage canopy where they encounter a complex matrix of variables (Table 3). Nevertheless, drawing broader conclusions would require further investigation involving non-RB viral strains and additional viral and fungal virus species using this unique experimental system.

Without fungal host resistance, systemic virus infection preceding fungus spray inoculation with different fungal RB and non-RB strain combinations only led to a small increase in fungal disease severity (up to 25%). By contrast, when fungal disease resistance was present, virus infection led to resistance breakdown of considerable magnitude. When the non-RB fungal strain was used alone and PGR was present, although it failed to elicit any fungal disease when virus infection was absent, it succeeded when virus infection was present. With the other four fungal RB and non-RB strain combinations, resistance breakdown was up to 7.4-fold with SDGR and 3.7-fold with PGR. The only time fungal host resistance was broken when virus infection was absent was when the fungal RB strain was used alone and SDGR was present. With systemically virus-infected but not virus mock-inoculated plants, mixing RB and non-RB strains into a combined inoculum resulted in the most severe fungal disease compared to inoculation with paired fungal strain combinations where its RB or non-RB strains were inoculated alone or in different first-last combinations. This is not unexpected, as inoculating plants with RB and non-RB plant pathogen strain mixtures often results in interactions that further diminish the overall efficiency of host plant resistance due to factors such as resistance breakdown, enhanced disease severity, intra-strain competition and reduced RB strain fitness [34]. When Abidin et al. [9] used fungal spore droplet inoculation onto virus-infected cotyledons of the same canola cultivars also used here, this failed to cause major resistance breakdown of fungal SDGR or PGR. This contrasts with the findings in the current study where whole plant spray inoculation onto virus-infected foliage achieved this in the presence of the same two fungal resistances. Spray inoculation typically causes more uniform and severe symptoms than a single-droplet application by substantially increasing the total surface area infected and the effective overall inoculum load [35,36,37]. This facilitates more rapid lesion coalescence and helps overcome localized host defenses. Thus, the combination of spray inoculation with virus infection enhanced the breakdown of SDGR and PGR. Although callose deposition was not investigated here, our spray inoculation procedure might also have contributed to the widespread fungal symptom development because fungal spore droplet inoculation stimulates higher callose deposition than spray inoculation [37].

Fungal inoculation onto systemically virus-infected plant foliage never caused breakdown of viral TSSIR. This is because VC values diminished (up to 72%) when TSSIR and fungal PGR were both present in coinfected plants. VC values also decreased (up to 49%) when fungal SDGR was present and when fungal resistance was absent (up to 30%). The magnitude of VC suppression in coinfected plants reflected fungal disease symptom severity, with this always being the greatest with the non-RB and RB strain inoculum mixture combination. When Abidin et al. [9] inoculated the same two fungal RB and non-RB strains alone onto entire virus-infected cotyledons or the non-RB strain onto half-cotyledons of the same three cultivars, there was marked VC suppression by both strains in young systemically infected leaves when fungal resistance was absent, but less suppression when SDGR or PGR + TSSIR were present or cotyledon halves were inoculated with the RB strain. This reflected the greater relative fungal disease symptom severity where no fungal resistance was operating, or the non-RB strain partially overcame resistance when absent from the other half-cotyledon. Partial breakdown of TSSIR due to the remote influence of fungal disease on cotyledons was evident because VC values were minimal in systemically virus-infected leaves on plants with PGR + TSSIR but without fungus-inoculated cotyledons. This difference between the two studies likely reflects remote influencing from localized fungal infection derived from droplet inoculation on cotyledons versus extensive fungal disease on coinfected leaves resulting from much more potent spray inoculation.

Inoculating spores of fungal pathogens onto virus-infected foliage of plant cultivars carrying fungal resistances is likely to cause fungal host resistance breakdown by weakening the plant’s overall defense system [38]. Such activity would be driven mainly by host defense signaling suppression accompanied by generation of conditions conducive to fungal colonization. Pre-existing viral infection would be likely to disrupt phytohormone signaling, specifically weakening jasmonic acid pathways and triggering anti-fungal defense inhibition [39,40]. Mechanisms that might enable this to occur include: (i) virus-induced hormonal trade-offs via jasmonic acid/salicylic acid “cross-talk” combined with phytoalexin and defensive enzyme suppression [41,42], (ii) virus-induced tissue damage and nutrient leaking facilitating fungal entry and nutrition [43] and (iii) system overload where resources are prioritized for the established virus over the invading fungus [42,44,45]. Furthermore, callose provides a critical physical barrier that reinforces the plant cell wall at pathogen attack sites [46]. This process involves formation of circular structures surrounding fungal infection foci, a feature absent in susceptible cultivars [46]. Viral pre-infection might compromise such protection when viral proteins activate the MAPK pathway to downregulate callose deposition [47]. Such barrier suppression would facilitate more extensive fungal penetration and subsequent colonization [46,47]. To verify if prior viral exposure shifts host immunity toward salicylic acid (SA) pathway activation at the expense of jasmonic acid (JA) signaling, future experiments should measure the transcript levels of classic marker genes, such as PR1 and PDF1.2, via qPCR or Northern blot analysis [48,49,50]. Furthermore, future studies should examine whether viral infection reduces callose deposition at plasmodesmata [47,51], alters MAPK cascade activation [47,52], modulates defense gene expression, or affects phytoalexin accumulation [53].

Although the fungal spray inoculation to plant foliage we used here better mimics natural infection than droplet inoculation to cotyledons, this study was still restricted to controlled environment glasshouse conditions and the use of small pots. Therefore, apart from investigating the mechanism by which previous virus presence impairs the effectiveness of fungal disease resistance, future research is needed to validate how prior viral infection impacts fungal pathogen resistance and how fungal coinfection influences viral resistance and VC. Specifically, future studies should investigate: (i) whether these reciprocal interactions also occur under field conditions where environmental variability, natural inoculum pressure, timing of infections, the chronology of infection and other mixed-pathogen populations all influence disease outcomes [6,7]; (ii) whether using different strain combinations of these pathogens or entirely different virus–fungus combinations results in similar resistance breakdowns across diverse host–pathogen resistances [9,54,55,56].

Despite being conducted under controlled glasshouse conditions, the findings of this study have broader implications for managing viral and fungal disease outbreaks across crop, pasture, and wild plant ecosystems [6,7]. Single-pathogen host resistance, whether deliberately bred into agricultural crops and pastures or occurring naturally in wild populations, is vulnerable to breakdown when host plants encounter co-infecting pathogens [9,56]. In the present study, prior viral infection compromised host resistance to a subsequent fungal pathogen. This lack of durability in single-pathogen resistance during mixed-pathogen epidemics presents serious challenges for plant breeding programs that rely exclusively on single-disease resistance without pyramiding genes against other co-occurring, damaging pathogens [57,58]. Furthermore, these findings underscore the risk of relying solely on host resistance, emphasizing the necessity of integrated disease management strategies that pair single-pathogen resistance with complementary phytosanitary, cultural, biological, and chemical control measures [59,60,61,62].

## 5. Conclusions

This research addresses an important and relatively underexplored aspect of plant pathology: viral–fungal pathogen interactions and their effects on pathogen resistance durability. It provides new insights into the risks of relying on single-pathogen plant resistance by demonstrating that previous virus infection can enhance host plant susceptibility to fungal disease under different host resistance backgrounds. These effects varied depending upon the specific resistance profile of the cultivated plant cultivar and whether the pathogen strains present were resistance-breaking or non-resistance-breaking. This research also highlights the importance of studying infections with co-occurring pathogens using a methodology that reflects real-world field situations. The approach adopted can serve as a foundational initial model system for developing an improved understanding of critical fungal and viral inter-pathogen and intra-pathogen interactions relevant to situations where plants with different types of single-pathogen host resistance are present. It can also address the critical need to reconsider the importance of fungal and viral coinfection scenarios in cultivated and wild plants. This applies not only to the greater economic impacts upon yield and quality losses likely to arise when the effectiveness of single-pathogen host resistance against widespread mixed virus–fungus infections is compromised, but also to the need to breed new cultivated plant cultivars with effective host resistance to both pathogens in frequently occurring coinfection circumstance. Beyond commercial agriculture, protective strategies are also required to safeguard wild plant populations carrying natural pathogen resistances from resistance breakdown caused by co-occurring pathogens, especially when such populations are growing in endangered ecosystems.

## Figures and Tables

**Table 1:** Inoculation treatments in each experiment.

Treatment	Initial Cotyledon Inoculation	Subsequent Fungal Spray Inoculation	Same Day (0–0 dai) or Delayed (0–3 dai)
1	Mock-inoculated	Deionized water	0–0
2	Mock-inoculated	non-RB (1st)–non-RB (2nd)	0–0
3	Mock-inoculated	non-RB (1st)–RB (2nd)	0–0
4	Mock-inoculated	RB (1st)–non-RB (2nd)	0–0
5	Mock-inoculated	RB (1st)–RB (2nd)	0–0
6	Mock-inoculated	non-RB + RB (1st)–non-RB + RB (2nd)	0–0
**7**	**Virus-inoculated**	**Deionized water**	**0–0**
**8**	**Virus-inoculated**	**non-RB (1st)–non-RB (2nd)**	**0–0**
**9**	**Virus-inoculated**	**non-RB (1st)–RB (2nd)**	**0–0**
**10**	**Virus-inoculated**	**RB (1st)–non-RB (2nd)**	**0–0**
**11**	**Virus-inoculated**	**RB (1st)–RB (2nd)**	**0–0**
**12**	**Virus-inoculated**	**non-RB + RB (1st)–non-RB + RB (2nd)**	**0–0**
13	Mock-inoculated	Deionized water	0–3
14	Mock-inoculated	non-RB (1st)–non-RB (2nd)	0–3
15	Mock-inoculated	non-RB (1st)–RB (2nd)	0–3
16	Mock-inoculated	RB (1st)–non-RB (2nd)	0–3
17	Mock-inoculated	RB (1st)–RB (2nd)	0–3
18	Mock-inoculated	non-RB + RB (1st)–non-RB + RB (2nd)	0–3
**19**	**Virus-inoculated**	**Deionized water**	**0–3**
**20**	**Virus-inoculated**	**non-RB (1st)–non-RB (2nd)**	**0–3**
**21**	**Virus-inoculated**	**non-RB (1st)–RB (2nd)**	**0–3**
**22**	**Virus-inoculated**	**RB (1st)–non-RB (2nd)**	**0–3**
**23**	**Virus-inoculated**	**RB (1st)–RB (2nd)**	**0–3**
**24**	**Virus-inoculated**	**non-RB + RB (1st)–non-RB + RB (2nd)**	**0–3**

Each experimental treatment consisted of initial canola seedling cotyledon inoculation with virus resistance-breaking (RB) strain isolate 12.1 or virus mock-inoculation with healthy leaf sap, and subsequent foliage fungal spore spray inoculation with different combinations of non-resistance-breaking (non-RB) strain isolate P11 and resistance-breaking (RB) strain isolate UWA192 or a mixed non-RB and RB strain inoculum. Fungal spray inoculation was done twice on the same day (i.e., at 0–0 dai), or the second inoculation was delayed for 3 days (i.e., at 0–3 dai). The delay between cotyledon inoculation and first fungal inoculation was 50 days. Second fungal inoculation was on same day (0–0 dai) or after 3 days (0–3 dai). Deionized water = fungus mock-inoculation (i.e., no *L. maculans*). Orange backround without bold font = virus mock-inoculated, purple background with bold font = virus-inoculated.

**Table 2:** Fungal percentage leaf disease outcomes following cotyledon virus inoculation followed by true-leaf fungal inoculation.

Same-Day Second Fungal Inoculation (0–0 dai)								
Resistance (Cultivar)	Initial Cotyledon Inoculation	Subsequent Fungal Isolate Inoculation	%LDI	%LAD	%LAC	VC (Before)	VC (After)	VC (Newest Tip)
SDGR	Mock-inoculated	Deionized water	0	0	0	1.1	1.1	1.1
(Surpass 400)		non-RB + RB (1st)–non-RB + RB (2nd)	10.5 b	10.2 b	6.3 b	1.1	1.1	1.1
		non-RB (1st)–non-RB (2nd)	9.8 b	9.8 b	6.4 b	1.1	1.1	1.1
		non-RB (1st)–RB (2nd)	10.2 b	10.0 b	6.0 b	1.1	1.1	1.1
		RB (1st)–RB (2nd)	26.0 c	18.1 c	19.3 c	1.1	1.1	1.1
		RB (1st)–non-RB (2nd)	10.4 b	10.1 b	6.4 b	1.1	1.1	1.1
**SDGR**	**Virus-inoculated**	**Deionized water**	**0**	**0**	**0**	**25.9 b**	**27.1 h**	**27.4 h**
**(Surpass 400)**		**non-RB + RB (1st)–non-RB + RB (2nd)**	**39.3 g**	**40.7 e**	**39.2 e**	**25.7 b**	**15.3 e**	**15.3 e**
		**non-RB (1st)–non-RB (2nd)**	**33.3 d**	**33.9 d**	**30.6 d**	**26.0 b**	**22.7 g**	**26.1 g**
		**non-RB (1st)–RB (2nd)**	**32.8 d**	**34.1 d**	**30.4 d**	**25.9 b**	**15.2 e**	**14.9 e**
		**RB (1st)–RB (2nd)**	**33.5 d**	**34.0 d**	**32.1 d**	**26.1 b**	**15.4 e**	**15.0 e**
		**RB (1st)–non-RB (2nd)**	**32.9 d**	**34.4 d**	**30.0 d**	**25.8 b**	**15.4 e**	**14.9 e**
PGR, TSSIR	Mock-inoculated	Deionized water	0	0	0	1.1	1.1	1.1
(Scoop)		non-RB + RB (1st)–non-RB + RB (2nd)	11.5 b	11.7 b	10.4 b	1.1	1.1	1.1
		non-RB (1st)–non-RB (2nd)	0 a	0 a	0 a	1.1	1.1	1.1
		non-RB (1st)–RB (2nd)	11.3 b	11.7 b	10.2 b	1.1	1.1	1.1
		RB (1st)–RB (2nd)	11.3 b	11.2 b	10.9 b	1.1	1.1	1.1
		RB (1st)–non-RB (2nd)	11.5 b	11.1 b	10.0 b	1.1	1.1	1.1
**PGR, TSSIR**	**Virus-inoculated**	**Deionized water**	**0**	**0**	**0**	**14.4 a**	**14.6 d**	**14.6 d**
**(Scoop)**		**non-RB + RB (1st)–non-RB + RB (2nd)**	**42.3 f**	**39.9 e**	**37.0 e**	**14.0 a**	**7.8 a**	**7.8 a**
		**non-RB (1st)–non-RB (2nd)**	**36.9 e**	**34.8 d**	**30.3 d**	**14.2 a**	**8.3 c**	**8.2 c**
		**non-RB (1st)–RB (2nd)**	**35.1 d**	**34.2 d**	**31.2 d**	**14.0 a**	**8.3 c**	**8.1 c**
		**RB (1st)–RB (2nd)**	**37.6 e**	**34.9 d**	**31.4 d**	**14.2 a**	**8.0 b**	**7.9 b**
		**RB (1st)–non-RB (2nd)**	**34.4 de**	**34.4 d**	**32.6 d**	**14.1 a**	**8.1 b**	**7.9 b**
Absent	Mock-inoculated	Deionized water	0	0	0	1.1	1.1	1.1
(Westar)		non-RB + RB (1st)–non-RB + RB (2nd)	55.4 gh	50.2 f	47.0 f	1.1	1.1	1.1
		non-RB (1st)–non-RB (2nd)	56.0 gh	35.9 d	30.3 d	1.1	1.1	1.1
		non-RB (1st)–RB (2nd)	55.3 gh	35.7 d	30.3 d	1.1	1.1	1.1
		RB (1st)–RB (2nd)	57.0 gh	37.2 d	30.4 d	1.1	1.1	1.1
		RB (1st)–non-RB (2nd)	56.7 gh	35.7 d	31.2 d	1.1	1.1	1.1
**Absent**	**Virus-inoculated**	**Deionized water**	**0**	**0**	**0**	**31.2 c**	**32.5 i**	**32.6 i**
**(Westar)**		**non-RB + RB (1st)–non-RB + RB (2nd)**	**64.5 j**	**60.2 g**	**62.7 h**	**30.8 c**	**14.3 d**	**14.2 d**
		**non-RB (1st)–non-RB (2nd)**	**59.0 i**	**54.2 f**	**48.4 f**	**30.9 c**	**17.8 f**	**17.4 f**
		**non-RB (1st)–RB (2nd)**	**59.0 i**	**50.8 f**	**47.1 f**	**30.7 c**	**17.8 f**	**17.3 f**
		**RB (1st)–RB (2nd)**	**59.1 i**	**49.3 d**	**46.5 f**	**31.1 c**	**29.2 h**	**29.1 h**
		**RB (1st)–non-RB (2nd)**	**59.3 i**	**51.0 f**	**46.6 f**	**30.9 c**	**29.3 h**	**29.0 h**
		***Level of significance***	***<0.001***	***<0.001***	***<0.001***	***<0.001***	***<0.001***	***<0.001***
		**LSD_(0.05)_**	**3.85**	**4.91**	**5.20**	**0.43**	**0.23**	**0.22**
**Delayed Second Fungal Inoculation (0–3 dai)**								
**Resistance (Cultivar)**	**Initial Cotyledon Inoculation**	**Subsequent Fungal Isolate Inoculation**	**%LDI**	**%LAD**	**%LAC**	**VC (Before)**	**VC (After)**	**VC (Newest Tip)**
SDGR	Mock-inoculated	Deionized water	0	0	0	1.1	1.1	1.1
(Surpass 400)		non-RB + RB (1st)–non-RB + RB (2nd)	10.4 b	8.4 bc	9.3 bc	1.1	1.1	1.1
		non-RB (1st)–non-RB (2nd)	10.2 b	6.2 b	8.1 bc	1.1	1.1	1.1
		non-RB (1st)–RB (2nd)	10.0 b	6.0 b	5.0 b	1.1	1.1	1.1
		RB (1st)–RB (2nd)	36.6 e	21.5 e	26.9 e	1.1	1.1	1.1
		RB (1st)–non-RB (2nd)	15.7 c	12.7 c	11.0 bc	1.1	1.1	1.1
**SDGR**	**Virus-inoculated**	**Deionized water**	**0**	**0**	**0**	**26.0 b**	**27.1 n**	**27.4 n**
**(Surpass 400)**		**non-RB + RB (1st)–non-RB + RB (2nd)**	**65.0 i**	**62.5 i**	**57.8 h**	**26.2 b**	**13.3 e**	**13.2 e**
		**non-RB (1st)–non-RB (2nd)**	**52.4 gh**	**52.1 gh**	**45.0 fg**	**26.0 b**	**25.8 m**	**25.5 m**
		**non-RB (1st)–RB (2nd)**	**53.3 gh**	**52.9 gh**	**44.3 fg**	**26.3 b**	**15.2 h**	**15.0 h**
		**RB (1st)–RB (2nd)**	**53.0 gh**	**54.0 h**	**44.0 fg**	**26.2 b**	**15.3 h**	**15.0 h**
		**RB (1st)–non-RB (2nd)**	**53.2 gh**	**54.7 h**	**44.0 fg**	**26.1 b**	**15.3 h**	**15.1 h**
PGR, TSSIR	Mock-inoculated	Deionized water	0	0	0	1.1	1.1	1.1
(Scoop)		non-RB + RB (1st)–non-RB + RB (2nd)	18.3 cd	18.5 de	15.0 c	1.1	1.1	1.1
		non-RB (1st)–non-RB (2nd)	0 a	0 a	0 a	1.1	1.1	1.1
		non-RB (1st)–RB (2nd)	20 cd	17.0 d	15.5 c	1.1	1.1	1.1
		RB (1st)–RB (2nd)	20.7 cd	21.1 de	18.9 cd	1.1	1.1	1.1
		RB (1st)–non-RB (2nd)	22.9 d	22.6 e	18.9 cd	1.1	1.1	1.1
**PGR, TSSIR**	**Virus-inoculated**	**Deionized water**	**0**	**0**	**0**	**14.2 a**	**14.6 g**	**14.8 g**
**(Scoop)**		**non-RB + RB (1st)–non-RB + RB (2nd)**	**56.6 h**	**56.3 hi**	**55.8 h**	**14.0 a**	**4.1 a**	**4.0 a**
		**non-RB (1st)–non-RB (2nd)**	**42.2 f**	**43.4 f**	**42.1 f**	**14.0 a**	**6.3 c**	**6.0 c**
		**non-RB (1st)–RB (2nd)**	**43.0 f**	**43.1 f**	**41.0 f**	**13.9 a**	**6.4 c**	**6.1 c**
		**RB (1st)–RB (2nd)**	**50.2 g**	**48.5 g**	**48.8 g**	**14.2 a**	**5.4 b**	**5.4 b**
		**RB (1st)–non-RB (2nd)**	**50.5 g**	**48.4 g**	**48.6 g**	**14.2 a**	**5.5 b**	**5.4 b**
Absent	Mock-inoculated	Deionized water	0	0	0	1.1	1.1	1.1
(Westar)		non-RB + RB (1st)–non-RB + RB (2nd)	72.6 k	68.8 i	63.1 ij	1.1	1.1	1.1
		non-RB (1st)–non-RB (2nd)	56.7 h	48.0 g	47.3 fg	1.1	1.1	1.1
		non-RB (1st)–RB (2nd)	57.0 h	48.8 g	46.0 fg	1.1	1.1	1.1
		RB (1st)–RB (2nd)	56.5 h	47.9 g	48.4 fg	1.1	1.1	1.1
		RB (1st)–non-RB (2nd)	56.1 h	48.5 g	49.0 fg	1.1	1.1	1.1
**Absent**	**Virus-inoculated**	**Deionized water**	**0**	**0**	**0**	**30.9 c**	**32.5 n**	**32.6 n**
**(Westar)**		**non-RB + RB (1st)–non-RB + RB (2nd)**	**80.8 l**	**80.0 j**	**74.2 k**	**30.7 c**	**9.8 d**	**9.8 d**
		**non-RB (1st)–non-RB (2nd)**	**69.0 ij**	**68.6 i**	**67.3 ij**	**30.8 c**	**14.2 f**	**14.0 f**
		**non-RB (1st)–RB (2nd)**	**72.3 j**	**60.0 h**	**62.5 ij**	**30.7 c**	**14.3 f**	**14.0 f**
		**RB (1st)–RB (2nd)**	**72.2 j**	**62.7 h**	**66.0 ij**	**30.9 c**	**22.1 k**	**22.0 k**
		**RB (1st)–non-RB (2nd)**	**71.6 j**	**64.0 hi**	**59.2 hi**	**31.0 c**	**23.1 L**	**22.8 L**
		***Level of significance***	***<0.001***	***<0.001***	***<0.001***	***<0.001***	***<0.001***	***<0.001***
		**LSD_(0.05)_**	**5.01**	**4.88**	**6.68**	**0.39**	**0.19**	**0.22**

Fungal percentage leaf disease index (%LDI), percentage leaf area diseased (%LAD) and percentage leaf area collapsed (%LAC) outcomes from different treatments following initial cotyledon virus inoculation or virus mock-inoculation. Subsequent fungal foliage spray inoculation was done on the same day (0–0 dai) or delayed days (0–3 dai). Virus concentration (VC) for systemically virus-infected plants was measured as ELISA Optical Density (OD) *e^x^*-transformed A_405_ nm values. Cultivar resistance/susceptibility status: fungal resistance lacking—cv. Westar, fungal single dominant gene resistance (SDGR)—cv. Surpass 400 and combined fungal polygenic resistance (PGR) and virus temperature-sensitive systemic invasion resistance (TSSIR)—cv. Scoop. Fungal strains used were resistance-breaking (RB) and non-resistance-breaking (non-RB). Deionized water = fungal mock-inoculation. VC value (before) represents tip leaves at 45 days after virus cotyledon inoculation and 5 days before subsequent 1st fungal inoculation. VC value (after) represents tip leaves at 14 days after subsequent 2nd fungal inoculation. VC (newest tip) represents the VC value of new tip leaves too small to inoculate at the time of fungal inoculation. Fungal isolates used in the study: non-RB strain isolate P11, RB strain isolate UWA192, or mixture of fungal non-RB and RB isolate inoculum. Virus isolate used was RB strain isolate 12.1. Different letters are significantly different (at *p* < 0.05) based on Fisher’s protected LSD. Background colors distinguish data between cultivars with SDGR (blue), PGR + TSSIR (green) and without fungus resistance (orange). With Bold = virus-inoculated. Without bold = virus mock-noculated.

**Table 3:** Reasons why the approach adopted in this study is unique.

• Simulation of Natural Field Dispersal: Previous work [e.g., 9] relied upon localized fungal spore droplet inoculation onto cotyledons. This system uses spray inoculation onto true leaves which closely mimics how fungal spores spread naturally via wind and rain across the plant foliage canopy.
• Broad Surface Exposure: Spray inoculation exposes the entire plant foliage surface area to the fungal pathogen, reflecting true agricultural field dynamics where plants face diffuse, multi-point infection pressure rather than isolated point-source infection.
• Multi-Layered Host Resistance Profiling with Differential Host Genotypes: Rather than looking at a single type of resistance, this system systematically compares single dominant gene resistance (SDGR), polygenic resistance (PGR) and environment-dependent resistance (temperature-sensitive systemic invasion resistance—TSSIR), and fully susceptible controls (lacking fungal resistance) and explores the interplay between resistance mechanisms, allowing researchers to study how viral pre-infection impacts both more durable, multi-gene resistance (PGR) and single-gene resistance (SDGR).
• Strain-Level Complexity/Co-occurrence of Virulent and Avirulent Strains: The system incorporates both resistance-breaking (RB) and non-resistance-breaking (non-RB) strains for the fungus. This enables testing whether a viral RB strain can act as a “saboteur” to facilitate infection by a non-RB fungal strain that would normally be restricted by host defenses.
• Systemic versus Localized Infection: By testing foliage both with and without established systemic viral infection, the system isolates the effect of active systemic viral presence upon host anti-fungal defenses.
• Inoculation Timing: Spacing the secondary fungal inoculations (twice on the same day vs. 3 days apart) accounts for how the timing of secondary pathogen arrival influences disease outcomes.
• Bidirectional Inquiry/Dual Hypothesis Framework: Unlike typical coinfection studies that look at one pathogen’s effect upon another, this setup tests a reciprocal dynamic: whether viral infection breaks fungal resistance (SDGR/PGR) and whether secondary fungal infection can reciprocally break viral resistance (TSSIR).

## Data Availability

The original contributions presented in this study are included in the article. Further inquiries can be directed to the corresponding author.

## Supplementary Materials

The following supporting information can be downloaded at: https://www.mdpi.com/article/10.3390/pathogens15090973/s1, Tables S1–S5. Table S1: Captians: Statistical outcomes in relation to the fungal % leaf disease incidence (%LDI); Table S2: Statistical outcomes in relation to the fungal % leaf area diseased (%LAD); Table S3: Statistical outcomes in relation to the fungal % leaf area collapsed (%LAC); Table S4: Statistical outcomes in relation to the virus titer before fungal inoculation; Table S5: Statistical outcomes in relation to the virus titer after fungal inoculation. Footnotes for each Supplementary Table: d.f. = degrees of freedom; F-value = a test statistic that compares the variance between different groups of data to the variance within those groups; *p*-value = the probability of obtaining test results at least as extreme as the ones observed, assuming that the null hypothesis is completely true.
