# Supplementary material for: Previous Viral Infection Triggers Fungal Resistance Breakdown in Host Plants
Source: Pathogens. 2026 Sep 14;15(9):973. doi: 10.3390/pathogens15090973 (PMC13610602; doi:10.3390/pathogens15090973)
Supplement: Supplementary file 1 [file pathogens-15-00973-s001.zip › pathogens-4441406-supplementary.pdf]

Supplementary Table S1: Statistical outcomes in relation to the fungal % leaf disease incidence (%LDI)

**Source of variation**	**d.f.**	**F-value**	***P*-value**
Cultivar	2	3112	<.001
Mock/virus inoculated	1	1295	<.001
Fungal isolate combination	4	1559	<.001
Fungal inoculation time	1	690	<.001
Cultivar x Mock/virus inoculated	2	272	<.001
Cultivar x Fungal isolate combination	8	145	<.001
Mock/virus inoculated x Fungal isolate combination	4	79	<.001
Cultivar x Fungal inoculation time	2	9	<.001
Mock/virus inoculated x Fungal inoculation time	1	139	<.001
Fungal isolate combination x Fungal inoculation time	4	46	<.001
Cultivar x Mock/virus inoculated x Fungal isolate combination	8	25	<.001
Cultivar x Mock/virus inoculated x Fungal inoculation time	2	21	<.001
Cultivar x Fungal isolate combination x Fungal inoculation time	8	4	<.001
Mock/virus inoculated x Fungal isolate combination x Fungal inoculation time	4	13	<.001
Cultivar x Mock/virus inoculated x Fungal isolate combination x Fungal inoculation time	8	5	<.001

Abbreviations: d.f. = degrees of freedom, F-value = is a test statistic used to compare the variance between different groups of data to the variance within those groups, and *P* -value = the probability of obtaining test results at least as extreme as the ones observed assuming that the null hypothesis is completely true.

Supplementary Table S2: Statistical outcomes in relation to the fungal % leaf area diseased (%LAD)

**Source of variation**	**d.f.**	**F-value**	***P*-value**
Cultivar	2	1974	<.001
Mock/virus inoculated	1	919	<.001
Fungal isolate combination	4	653	<.001
Fungal inoculation time	1	378	<.001
Cultivar x Mock/virus inoculated	2	25	<.001
Cultivar x Fungal isolate combination	8	133	<.001
Mock/virus inoculated x Fungal isolate combination	4	38	<.001
Cultivar x Fungal inoculation time	2	17	<.001
Mock/virus inoculated x Fungal inoculation time	1	100	<.001
Fungal isolate combination x Fungal inoculation time	4	33	<.001
Cultivar x Mock/virus inoculated x Fungal isolate combination	8	17	<.001
Cultivar x Mock/virus inoculated x Fungal inoculation time	2	6	<0.05
Cultivar x Fungal isolate combination x Fungal inoculation time	8	13	<.001
Mock/virus inoculated x Fungal isolate combination x Fungal inoculation time	4	6	<.001
Cultivar x Mock/virus inoculated x Fungal isolate combination x Fungal inoculation time	8	8	<.001

Abbreviations: d.f. = degrees of freedom, F-value = is a test statistic used to compare the variance between different groups of data to the variance within those groups, *P* -value = the probability of obtaining test results at least as extreme as the ones observed assuming that the null hypothesis is completely true.

Supplementary Table S3 Statistical outcomes in relation to the fungal % leaf area collapsed (%LAC)

**Source of variation**	**d.f.**	**F-value**	***P*-value**
Cultivar	2	1197	<.001
Mock/virus inoculated	1	732	<.001
Fungal isolate combination	4	443	<.001
Fungal inoculation time	1	321	<.001
Cultivar x Mock/virus inoculated	2	32	<.001
Cultivar x Fungal isolate combination	8	72	<.001
Mock/virus inoculated x Fungal isolate combination	4	37	<.001
Cultivar x Fungal inoculation time	2	22	<.001
Mock/virus inoculated x Fungal inoculation time	1	15	<.001
Fungal isolate combination x Fungal inoculation time	4	26	<.001
Cultivar x Mock/virus inoculated x Fungal isolate combination	8	21	<.001
Cultivar x Mock/virus inoculated x Fungal inoculation time	2	6	<.05
Cultivar x Fungal isolate combination x Fungal inoculation time	8	3	<.001
Mock/virus inoculated x Fungal isolate combination x Fungal inoculation time	4	4	<.001
Cultivar x Mock/virus inoculated x Fungal isolate combination x Fungal inoculation time	8	8	<.001

d.f. = degrees of freedom; F-value = a test statistic that compares the variance between different groups of data to the variance within those groups; P-value = the probability of obtaining test results at least as extreme as the ones observed assuming that the null hypothesis is completely true.

Supplementary Table S4: Statistical outcomes in relation to the virus concentration before fungal inoculation

**Source of variation**	**d.f.**	**F-value**	***P*-value**
Cultivar	2	31970	<.001
Fungal isolate combination	4	4	<.05
Fungal inoculation time	1	4	<.05
Cultivar x Fungal isolate combination	8	1	>0.05
Cultivar x Fungal inoculation time	2	7	<.001
Fungal isolate combination x Fungal inoculation time	4	4	<.05
Cultivar x Fungal isolate combination x Fungal inoculation time	8	4	<.001

d.f. = degrees of freedom; F-value = a test statistic that compares the variance between different groups of data to the variance within those groups; P-value = the probability of obtaining test results at least as extreme as the ones observed assuming that the null hypothesis is completely true.

Supplementary Table S5: Statistical outcomes in relation to the virus concentration after fungal inoculation

**Source of variation**	**d.f.**	**F-value**	***P*-value**
Cultivar	2	1463	<.001
Fungal isolate combination	4	314	<.001
Fungal inoculation time	1	107	<.001
Cultivar x Fungal isolate combination	8	101	<.001
Cultivar x Fungal inoculation time	2	64	<.001
Fungal isolate combination x Fungal inoculation time	4	41	<.001
Cultivar x Fungal isolate combination x Fungal inoculation time	8	5	<.001

d.f. = degrees of freedom; F-value = a test statistic that compares the variance between different groups of data to the variance within those groups; P-value = the probability of obtaining test results at least as extreme as the ones observed assuming that the null hypothesis is completely true.
